# Effects of prior therapies on outcomes with trifluridine/tipiracil in patients with metastatic gastric/gastroesophageal junction cancer in a randomized phase III trial (TAGS)

**DOI:** 10.1007/s00432-023-04813-z

**Published:** 2023-05-22

**Authors:** Kohei Shitara, Ben George, Julien Taieb, Raghav Sundar, Marwan G. Fakih, Lukas Makris, Karim A. Benhadji, Michele Ghidini

**Affiliations:** 1 National Cancer Center Hospital East, Kashiwa-Shi, Chiba 277-8577 Japan; 2 Nagoya University Graduate School of Medicine, Nagoya, Japan; 3 Medical College of Wisconsin, Milwaukee, WI USA; 4 Hôpital Européen Georges Pompidou, Université Paris-Cité, SIRIC CARPEM, Paris, France; 5 National University Health System, Singapore, Singapore; 6 City of Hope Comprehensive Cancer Center, Duarte, CA USA; 7 Stathmi, Inc., New Hope, PA USA; 8 Taiho Oncology, Inc., Princeton, NJ USA; 9 Azienda Ospedaliera di Cremona, Cremona, Italy

**Keywords:** Gastric cancer, Gastroesophageal junction cancer, Trifluridine tipiracil, Antineoplastic protocols, Third- or later-line therapy

## Abstract

**Background:**

In the phase III TAGS trial, trifluridine/tipiracil showed survival benefit versus placebo in patients with metastatic gastric/gastroesophageal junction cancer and ≥ 2 prior chemotherapies. This post hoc exploratory analysis assessed the impact of prior therapy type on outcomes.

**Methods:**

Based on prior treatment, patients in TAGS (*N* = 507) were categorized into overlapping subgroups: ramucirumab ± other agents (*n* = 169), no ramucirumab (*n* = 338), paclitaxel but no ramucirumab (*n* = 136), ramucirumab + paclitaxel sequentially or in combination (*n* = 154), neither paclitaxel nor ramucirumab (*n* = 202), irinotecan (*n* = 281), and no irinotecan (*n* = 226). Overall and progression-free survival, time to Eastern Cooperative Oncology Group performance status (ECOG PS) ≥ 2, and safety were assessed.

**Results:**

Baseline characteristics and prior therapy patterns were generally well balanced between trifluridine/tipiracil and placebo arms across subgroups. Trifluridine/tipiracil was associated with survival benefits versus placebo regardless of prior treatment: across subgroups, median overall survival was 4.6–6.1 versus 3.0–3.8 months (hazard ratios, 0.47–0.88), median progression-free survival was 1.9–2.3 versus 1.7–1.8 months (hazard ratios, 0.49–0.67), and median time to ECOG PS ≥ 2 was 4.0–4.7 versus 1.9–2.5 months (hazard ratios, 0.56–0.88). Among trifluridine/tipiracil-randomized patients, median overall and progression-free survival trended longer in those who had not received ramucirumab, paclitaxel and ramucirumab, or irinotecan (6.0–6.1 and 2.1–2.3 months, respectively) than in those who previously received these agents (4.6–5.7 and 1.9 months). The trifluridine/tipiracil safety profile was consistent across subgroups, with similar overall incidences of grade ≥ 3 adverse events. Minor variations in hematologic toxicities were noted.

**Conclusions:**

In TAGS, third- or later-line trifluridine/tipiracil treatment demonstrated overall and progression-free survival and functioning benefits versus placebo and a consistent safety profile in patients with metastatic gastric/gastroesophageal junction cancer, regardless of prior treatment type.

**Clinical trials registration:**

clinicaltrials.gov NCT02500043.

**Supplementary Information:**

The online version contains supplementary material available at 10.1007/s00432-023-04813-z.

## Introduction

Patients with metastatic gastric or gastroesophageal junction cancer (mGC/GEJC) generally have a poor prognosis (Hsu et al. 2020), and only modest increases in overall survival (OS) are reported with first- and second-line therapies. In the first-line setting, the recommended treatment for advanced metastatic disease includes a platinum and fluoropyrimidine doublet, with trastuzumab for patients with human epidermal growth factor 2 [HER2]-positive GC. Recently, the immune checkpoint inhibitors nivolumab and pembrolizumab have shown promise in combination with chemotherapy or trastuzumab or both in the first-line setting (Janjigian et al. 2020; Janjigian 2021a, 2020b; Shitara 2020a). Nivolumab plus chemotherapy improved OS compared to chemotherapy alone in patients with GC with a PD-L1 combined positive score more than 5 and has become standard treatment in available countries (Janjigian 2021a). Pembrolizumab in combination with trastuzumab and chemotherapy improved response rates in HER2-positive GC (Janjigian et al. 2021b) and was granted accelerated FDA approval. Taxanes, irinotecan (IRI), and the vascular endothelial growth factor receptor 2 antibody ramucirumab (RAM) are generally recommended for second-line treatment (Smyth et al. 2020). Trastuzumab deruxtecan was approved in the US and Japan for previously treated HER2-positive GC after trastuzumab (Shitara et al. 2020b).

Disease progression is common after first- and second-line treatments in patients with mGC/GEJC, necessitating viable third-line treatment options for these patients (Chrom et al. 2015). Nivolumab and pembrolizumab monotherapy has shown benefit in previously treated patients with GC/GEJC (Baxter et al. 2021; Takei et al. 2022; Kang et al. 2017; Shitara et al. 2018a); although nivolumab is approved in Asia, the approval for pembrolizumab was recently withdrawn (Merck.com 2022). Trifluridine and tipiracil (FTD/TPI; TAS-102), comprising trifluridine, a thymidine analog, and tipiracil, a thymidine phosphorylase inhibitor (Emura et al. 2005; Lenz et al. 2015), was approved for third- or later-line treatment of patients with mGC/GEJC based on results from the randomized, double-blind, placebo-controlled, phase III trial (TAGS) (Shitara et al. 2018b). In TAGS, FTD/TPI significantly increased OS, the primary endpoint, versus placebo (median, 5.7 versus 3.6 months; hazard ratio [HR], 0.69 [95% confidence interval (CI), 0.56–0.85]; *p* = 0.00058), as well as progression-free survival (PFS; median 2.0 versus 1.8 months; HR, 0.57; 95% CI, 0.47–0.70; *p* < 0.0001) in 507 patients with mGC/GEJC who had received ≥ 2 prior chemotherapy regimens (Shitara et al. 2018b; Ilson et al. 2020; Mansoor et al. 2021). FTD/TPI also demonstrated a manageable safety profile, with hematologic and gastrointestinal-related adverse events (AEs) being the most common (Shitara et al. 2018b).

The TAGS population had received ≥ 2 standard-of-care regimens for advanced disease, and there was no upper limit on the number of prior therapies a patient could have received; in addition, the prior therapy sequence was not specified (Shitara et al. 2018b). Although multiple treatment options are available for patients with mGC/GEJC, no clear standard of care exists for the different lines of therapy, and sequencing of treatment is not addressed in current guidelines (Catenacci et al. 2021). Few reports exist on whether prior therapy affects outcome of later-line treatment, and data from these reports are mixed. Sub-analyses of the ATTRACTION-2 trial showed that previous therapy with either trastuzumab or RAM did not significantly affect the efficacy of later-line nivolumab in Japanese patients, but numerically higher objective response rates and PFS were observed in patients who previously received RAM (Kato et al. 2019; Satoh et al. 2020). A retrospective study of Korean patients determined that the sequence of taxane and IRI therapies in second and third lines did not affect OS in patients who received fluoropyrimidine and platinum first-line treatment (Choi et al. 2018). In contrast, two studies suggested that prior anti–PD-1 therapy improved the outcomes associated with subsequent taxane plus RAM treatment (Sasaki et al. 2020; Kankeu Fonkoua et al. 2021). Additional analyses are needed to clarify the expected treatment effect of FTD/TPI based on the previous therapies for mGC/GEJC.

In this analysis, we examined whether prior treatment with RAM, RAM plus paclitaxel (PAC), or IRI, standard second-line treatments for mGC/GEJC, influenced efficacy or safety outcomes in the phase III TAGS trial.

## Methods

### Study design and treatment

The design of the randomized, double-blind, placebo-controlled, phase III TAGS study (NCT02500043; registered on July 16, 2015), which was performed in 110 academic hospitals across 17 countries, has been previously described in detail (Shitara et al. 2018b). The TAGS study was performed in accordance with Declaration of Helsinki and Good Clinical Practice Guidelines specified by the International Conference on Harmonisation. Prior to study initiation, the protocol and all amendments were approved by the institutional review board or independent ethics committee at each participating site. Written, informed consent was obtained from all enrolled patients.

Patients were randomized in a 2:1 ratio (stratified by geographic region, Eastern Cooperative Oncology Group performance status (ECOG PS), and prior RAM treatment) to receive either oral FTD/TPI 35 mg/m^2^ twice daily on days 1–5 and days 8–12 every 28 days plus best supportive care (BSC) or placebo plus BSC. Treatment continued until disease progression, intolerance, or patient withdrawal.

### Patients

Eligible patients were aged ≥ 18 years (≥ 20 years in Japan); had an ECOG PS of 0–1; had histologically confirmed, non-resectable, mGC/GEJC; and had previously received ≥ 2 standard-of-care regimens for advanced disease.

Previous regimens must have included a fluoropyrimidine; a platinum agent; and a taxane, IRI, or both. Patients with HER2-positive tumors must have received prior anti-HER2 therapy, if available. Adjuvant therapy could be regarded as one previous regimen in patients who had recurrence during or within 6 months of postoperative adjuvant completion. In cases of preoperative and postoperative adjuvant chemotherapy, adjuvant therapy could be regarded as one previous regimen only if the same regimen was administered both preoperatively and postoperatively. Patients with recurrent disease were eligible providing that they had received ≥ 2 lines of chemotherapy.

### Endpoints and assessments

The primary endpoint of the TAGS study was OS (defined as the time from randomization to death). Key secondary endpoints were investigator-assessed PFS (defined as the time from randomization to radiological disease progression, per RECIST v1.1, or death), time to ECOG ≥ 2, and safety and tolerability.

Tumor assessments of the chest, abdomen, and pelvis (as clinically indicated) were performed within 28 days prior to treatment initiation and every 8 weeks thereafter until radiological disease progression, death, or the initiation of subsequent antineoplastic therapy. Safety and tolerability were assessed based the incidence of AEs (graded per National Cancer Institute Common Terminology Criteria for Adverse Events v4.03) that occurred between the provision of informed consent and 30 days after the final dose of study treatment.

### Statistical analysis

The main statistical considerations for the primary analysis have been reported previously (Shitara et al. 2018b). In the present analysis, efficacy and safety outcomes were evaluated based on prior treatment, and patients were categorized into subgroups based on prior treatment received: (1) prior RAM (alone or in combination with other agents); (2) no RAM or RAM-containing regimens; (3) prior PAC, but no RAM; (4) both PAC and RAM (sequentially or in combination); (5) neither PAC nor RAM; (6) prior IRI or IRI-containing regimens; and (7) no IRI or IRI-containing regimens. These patient subgroups were overlapping (nonexclusive), and any patient could be categorized into one or more subgroups. The first two subgroups (RAM and no RAM) were prespecified; the remainder were evaluated post hoc.

All randomized patients (intention-to-treat population) were included in efficacy analyses, and all patients who had received ≥ 1 dose of study treatment (as-treated population) were included in the safety analysis.

Comparisons of OS, PFS, and time to ECOG ≥ 2 between treatment groups in each subgroup were conducted using the stratified log-rank test, with median values calculated using the Kaplan–Meier method and associated HRs and 95% CIs calculated using a Cox proportional hazards model; *p* values will not be reported because of the exploratory nature of this analysis. Given that the subgroup analyses were not powered for statistical significance, no formal comparisons between the prior therapy subgroups were performed.

## Results

### Patient population

Between February 24, 2016, and January 5, 2018, 507 patients were enrolled and randomized to either the FTD/TPI (*n* = 337) or placebo (*n* = 170) arms and 503 patients (335 in the FTD/TPI arm and 168 in the placebo arm) received ≥ 1 dose of study treatment (Shitara et al. 2018b). Among these 507 patients, 33% had received prior RAM, 27% had received PAC (but no RAM), 30% had received both PAC and RAM, and 55% had received IRI. Only 3% of all patients received prior RAM without receiving prior PAC (not included in this analysis). Overall, the patterns of prior therapy received were balanced between the treatment arms (Supplementary Fig. 1).

Table 1 shows the distribution of baseline characteristics across the prior therapy subgroups. No appreciable differences were noted in baseline demographics. Across subgroups, most patients were male (69–78%), had mGC (65–76%), and had an ECOG PS of 1 (61–65%). Most patients were European (57–96%) and White (53–85%). The mean age ranged from 62 to 64 years. Although HER2-negative disease appeared to be predominant across all subgroups (51–70% of patients), high proportions of unavailable data prevented the classification of a substantial proportion of patients (6–30%) by HER2 status. Variations were noted in prior therapy patterns across regions: among patients in Japan (*n* = 73), the majority had previously received RAM (78%) and/or IRI (79%), and only a minority had received PAC without RAM (16%). In contrast, 24% of patients in Europe (*n* = 408) had previously received RAM, and 50% had previously received IRI.

**Table 1:** Baseline demographics and disease characteristics by prior treatment (ITT population)

Characteristic	RAM		No RAM		PAC (no RAM)		RAM + PAC		No RAM or PAC		IRI		No IRI	
	FTD/	Placebo	FTD/	Placebo	FTD/	Placebo	FTD/	Placebo	FTD/	Placebo	FTD/	Placebo	FTD/	Placebo
	TPI		TPI		TPI		TPI		TPI		TPI		TPI	
	(*n* = 114)	(*n* = 55)	(*n* = 223)	(*n* = 115)	(*n* = 99)	(*n* = 37)	(*n* = 106)	(*n* = 48)	(*n* = 124)	(*n* = 78)	(*n* = 183)	(*n* = 98)	(*n* = 154)	(*n* = 72)
Age														
Median (range), years	65 (37–81)	65 (42–80)	63 (24–89)	61 (32–82)	63 (27–89)	63 (39–79)	65 (37–81)	66 (42–80)	64 (24–83)	60 (32–82)	64 (24–83)	64 (39–82)	63 (38–89)	61 (32–79)
≥ 65 y, *n* (%)	60 (53)	31 (56)	94 (42)	43 (37)	41 (41)	17 (46)	56 (53)	26 (54)	53 (43)	26 (33)	87 (48)	47 (48)	67 (44)	27 (38)
Sex, *n* (%)														
Male	83 (73)	41 (75)	169 (76)	76 (66)	80 (81)	26 (70)	78 (74)	37 (77)	89 (72)	50 (64)	136 (74)	73 (74)	116 (75)	44 (61)
Race, *n* (%)														
White	68 (60)	22 (40)	176 (79)	91 (79)	75 (76)	32 (87)	64 (60)	19 (40)	101 (82)	59 (76)	111 (61)	53 (54)	133 (86)	60 (83)
Asian	35 (31)	24 (44)	16 (7)	5 (4)	10 (10)	3 (8)	32 (30)	20 (42)	6 (5)	2 (3)	38 (21)	24 (24)	13 (8)	5 (7)
Black/African American	1 (1)	2 (4)	0	0	0	0	0	2 (4)	0	0	1 (1)	2 (2)	0	0
Other	1 (1)	1 (2)	2 (1)	1 (1)	2 (2)	0	1 (1)	1 (2)	0	1 (1)	1 (1)	1 (1)	2 (1)	1 (1)
Unknown/missing	9 (8)	6 (11)	29 (13)	18 (16)	12 (12)	2 (5)	9 (8)	6 (13)	17 (14)	16 (21)	32 (18)	18 (18)	6 (4)	6 (8)
Geographic region, *n* (%)														
Europe	67 (59)	29 (53)	203 (91)	109 (95)	85 (86)	34 (92)	64 (60)	25 (52)	118 (95)	76 (96)	136 (74)	70 (71)	134 (87)	68 (94)
Japan	34 (30)	23 (42)	12 (5)	4 (4)	9 (9)	3 (8)	31 (29)	20 (42)	3 (2)	1 (1)	35 (19)	23 (24)	11 (7)	4 (6)
USA	13 (11)	3 (6)	8 (4)	2 (2)	5 (5)	0	11 (10)	3 (6)	3 (2)	2 (3)	12 (7)	5 (5)	9 (6)	0
ECOG PS, *n* (%)														
0	42 (37)	24 (44)	81 (36)	44 (38)	33 (33)	14 (38)	37 (35)	21 (44)	48 (39)	30 (38)	63 (34)	40 (41)	60 (39)	28 (39)
1	72 (63)	31 (56)	142 (64)	71 (62)	66 (67)	23 (62)	69 (65)	27 (56)	76 (61)	48 (62)	120 (66)	58 (59)	94 (61)	44 (61)
Primary tumor location, *n* (%)														
Gastric	80 (70)	38 (69)	159 (71)	83 (72)	65 (66)	24 (65)	73 (69)	32 (67)	94 (76)	59 (76)	130 (71)	70 (71)	109 (71)	51 (71)
GEJ	34 (30)	17 (31)	64 (29)	30 (26)	34 (34)	11 (30)	33 (31)	16 (33)	30 (24)	19 (24)	53 (29)	27 (28)	45 (29)	20 (28)
Both	0	0	0	2 (2)	0	2 (5)	0	0	0	0	0	1 (1)	0	1 (1)
Peritoneal Metastases,* n* (%)														
Yes	33 (29)	14 (25)	54 (24)	39 (34)	23 (23)	13 (35)	32 (30)	10 (21)	31 (25)	26 (33)	55 (30)	31 (32)	32 (21)	22 (31)
No	81 (71)	41 (75)	169 (76)	76 (66)	76 (77)	24 (65)	74 (70)	38 (79)	93 (75)	52 (67)	128 (70)	67 (68)	122 (79)	50 (69)
HER2 status, *n* (%)														
Positive	31 (27)	16 (29)	36 (16)	11 (10)	24 (24)	5 (14)	30 (28)	14 (29)	12 (10)	6 (8)	34 (19)	17 (17)	33 (21)	10 (14)
Negative	75 (66)	37 (67)	132 (59)	69 (60)	57 (58)	21 (57)	68 (64)	32 (67)	75 (60)	48 (62)	127 (69)	70 (71)	80 (52)	36 (50)
Unavailable	8 (7)	2 (4)	54 (24)	35 (30)	17 (17)	11 (30)	8 (8)	2 (4)	37 (30)	24 (31)	21 (12)	11 (11)	41 (27)	26 (36)
Number of metastatic sites, *n* (%)														
1–2	55 (48)	20 (36)	100 (45)	52 (45)	47 (48)	15 (41)	51 (48)	17 (35)	53 (43)	37 (47)	77 (42)	38 (39)	78 (51)	34 (47)
≥ 3	59 (52)	35 (64)	123 (55)	63 (55)	52 (53)	22 (60)	55 (52)	31 (65)	71 (57)	41 (53)	106 (58)	60 (61)	76 (49)	38 (53)

*ECOG PS* Eastern Cooperative Oncology Group performance status, *FTD/TPI* trifluridine/tipiracil, *GEJ* gastroesophageal junction, *HER2* human epidermal growth factor receptor 2, *IRI* irinotecan, *ITT* intent-to-treat, *PAC* paclitaxel, *RAM* ramucirumab

Table 2 shows details of prior therapy across the subgroups. Whereas prior gastrectomy or radiotherapy was well balanced across subgroups, the number of prior treatment regimens varied, with relatively few patients in the no RAM, PAC without RAM, no RAM or PAC, and no IRI subgroups reporting ≥ 4 prior treatment regimens compared with the RAM, RAM plus PAC, and IRI subgroups (9%–21% versus 36%–44%). Overall, nearly all patients had received fluoropyrimidines and/or platinum-containing agents as systemic therapy for metastatic disease. The use of prior anti–PD1/PD-L1 agents was low (3–11%) across all subgroups. Except for patients in the PAC without RAM (42%) and no IRI (0%) subgroups, most patients reported prior treatment with IRI (51–100%).

**Table 2:** Details of prior therapies in the prior therapy subgroups (ITT population)

Prior therapy type	RAM		No RAM		PAC (no RAM)		RAM + PAC		No RAM or PAC		IRI		No IRI	
	FTD/	Placebo	FTD/	Placebo	FTD/	Placebo	FTD/	Placebo	FTD/	Placebo	FTD/	Placebo	FTD/	Placebo
	TPI		TPI		TPI		TPI		TPI		TPI		TPI	
	(*n* = 114)	(*n* = 55)	(*n* = 223)	(*n* = 115)	(*n* = 99)	(*n* = 37)	(*n* = 106)	(*n* = 48)	(*n* = 124)	(*n* = 78)	(*n* = 183)	(*n* = 98)	(*n* = 154)	(*n* = 72)
Prior gastrectomy, *n* (%)														
Yes	57 (50)	26 (47)	90 (40)	48 (42)	42 (42)	19 (51)	53 (50)	23 (48)	48 (39)	29 (37)	80 (44)	43 (44)	67 (44)	31 (43)
Prior radiotherapy, *n* (%)														
Yes	26 (23)	13 (24)	45 (20)	13 (11)	26 (26)	5 (14)	23 (22)	12 (25)	19 (15)	8 (10)	45 (25)	16 (16)	26 (17)	10 (14)
Number of prior treatment regimens, *n* (%)														
2	28 (25)	8 (15)	98 (44)	56 (49)	30 (30)	15 (41)	27 (26)	8 (17)	68 (55)	41 (53)	29 (16)	19 (19)	97 (63)	45 (62)
3	39 (34)	19 (35)	95 (43)	41 (36)	48 (48)	14 (38)	37 (35)	15 (31)	47 (38)	27 (35)	94 (51)	38 (39)	40 (26)	22 (31)
≥ 4	47 (41)	28 (51)	30 (14)	18 (16)	21 (21)	8 (22)	42 (40)	25 (52)	9 (7)	10 (13)	60 (33)	41 (42)	17 (11)	5 (7)
Prior systemic regimens for metastatic cancer, *n* (%)^a^														
Fluoro-pyrimidines^b^	107 (94)	53 (96)	220 (99)	110 (96)	98 (99)	33 (89)	100 (94)	47 (98)	122 (98)	77 (99)	181 (99)	93 (95)	146 (95)	70 (97)
Platinum-containing compounds^c^	103 (90)	54 (98)	214 (96)	106 (92)	95 (96)	32 (86)	95 (90)	48 (100)	119 (96)	74 (95)	171 (93)	91 (93)	146 (95)	70 (97)
Taxanes^d^	112 (98)	54 (98)	194 (87)	92 (80)	99 (100)	37 (100)	106 (100)	48 (100)	95 (77)	55 (71)	155 (85)	74 (76)	151 (98)	72 (100)
Irinotecan^e^	112 (98)	54 (98)	113 (51)	59 (51)	42 (42)	15 (41)	63 (59)	35 (73)	71 (57)	44 (56)	183 (100)	98 (100)	0	0
HER2-inhibitors^f^	29 (25)	14 (26)	30 (14)	9 (8)	22 (22)	4 (11)	28 (26)	13 (27)	8 (6)	5 (6)	30 (16)	15 (15)	29 (19)	8 (11)
Immuno-therapy	14 (12)	5 (9)	11 (5)	2 (2)	6 (6)	1 (3)	13 (12)	3 (6)	5 (4)	1 (1)	17 (9)	4 (4)	8 (5)	3 (4)
Other	30 (26)	11 (20)	46 (21)	27 (24)	21 (21)	11 (30)	28 (26)	8 (17)	25 (20)	16 (21)	42 (23)	15 (15)	34 (22)	23 (32)

*FTD/TPI* trifluridine/tipiracil, *HER2* human epidermal growth factor receptor 2, *IRI* irinotecan, *ITT* intent-to-treat, *PAC* paclitaxel, *RAM* ramucirumab

^a^Patients could have received more than one category of treatment

^b^Included 5-fluorouracil, capecitabine, doxifluridine, S-1, tegafur and uracil/tegafur, and agents collected as 'other' and later remapped to ‘fluoropyrimidines’

^c^Included oxaliplatin, cisplatin, carboplatin, and agents collected as 'other' and later remapped to ‘platinum-containing compounds’

^d^Included docetaxel, paclitaxel, nab-paclitaxel (abraxane), and agents collected as 'other' and later remapped to ‘taxanes’

^e^Included irinotecan, CPT-11, and agents collected as 'other' and later remapped to ‘irinotecan’

^f^Included trastuzumab, pertuzumab, and TDM-1

### Patient disposition

As described in the primary analysis, 94% of patients in the FTD/TPI arm and 98% of patients in the placebo arm had discontinued treatment by the data cutoff date (March 31, 2018; Supplementary Table 1) (Shitara et al. 2018b). Overall discontinuation rates of FTD/TPI-treated patients did not vary appreciably in the prior therapy subgroups (ranging from 90% in the no IRI subgroup to 98% in the IRI subgroup). FTD/TPI-treated patients who previously received RAM had higher rates of discontinuation due to clinical or radiological progression (91%) and lower AE-related discontinuations (2–3%) than other subgroups (discontinuations due to radiological/clinical progression, 59–77%; due to AEs, 9–15%).

### Efficacy

**Fig. 1:**
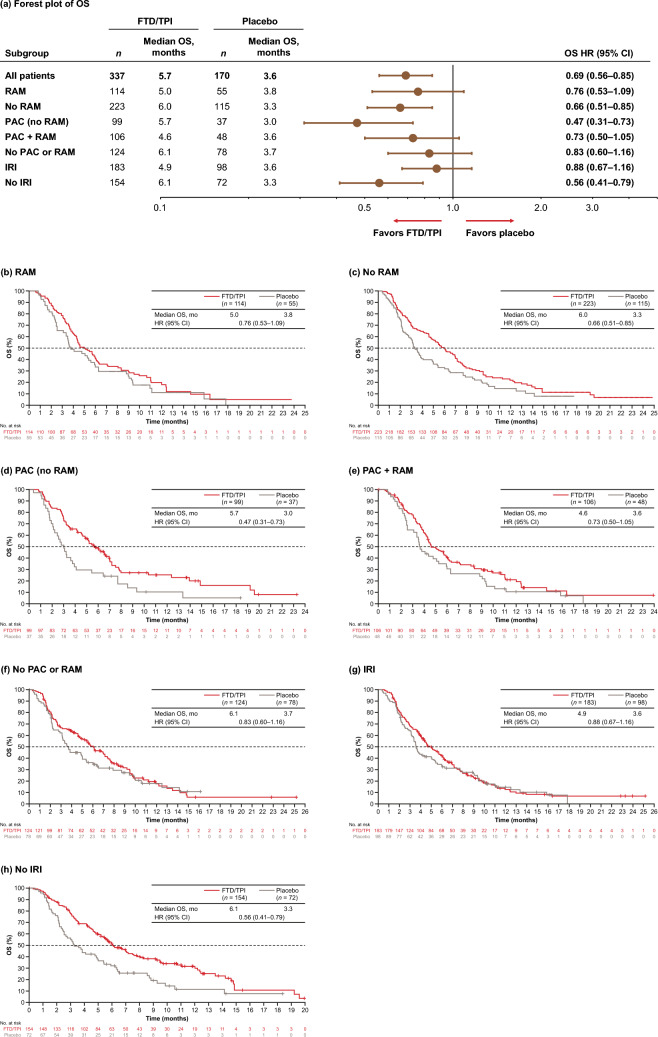
**a** Forest plot and **b**–**h** Kaplan–Meier plots of OS across prior treatment subgroups (ITT population). *CI* confidence interval, *FTD/TPI* trifluridine/tipiracil, *HR* hazard ratio, *IRI* irinotecan, *ITT* intent-to-treat, *OS* overall survival, *PAC* paclitaxel, *RAM* ramucirumab

**Fig. 2:**
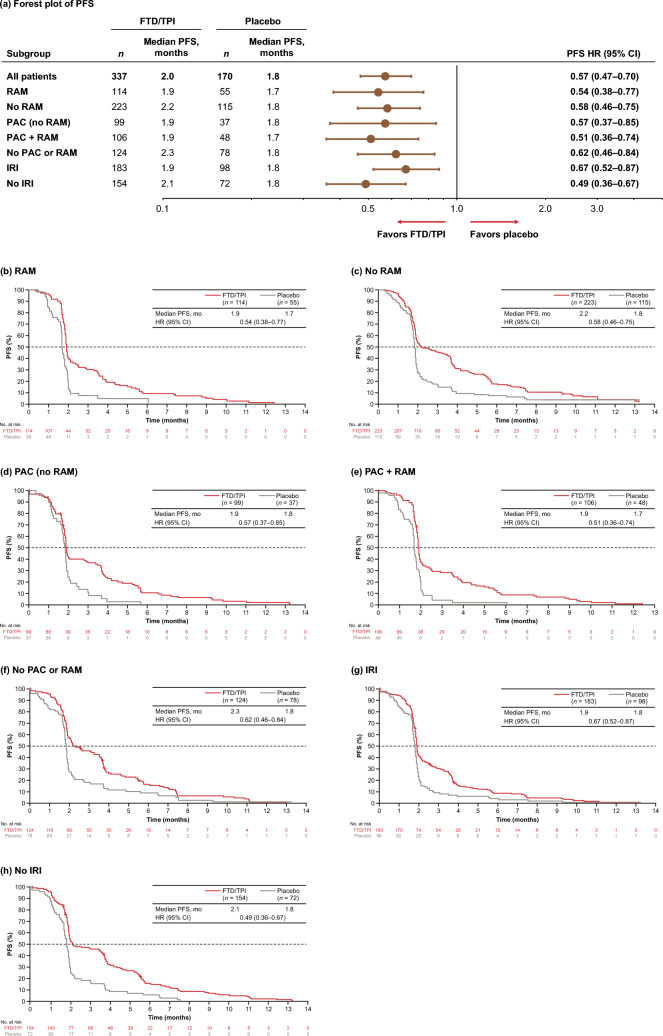
**a** Forest plot and **b**–**h** Kaplan–Meier plots of PFS across prior treatment subgroups (ITT population). *CI* confidence interval, *FTD/TPI* trifluridine/tipiracil, *HR* hazard ratio, *IRI* irinotecan, *ITT* intent-to-treat, *PAC* paclitaxel, *PFS* progression-free survival, *RAM* ramucirumab

**Fig. 3:**
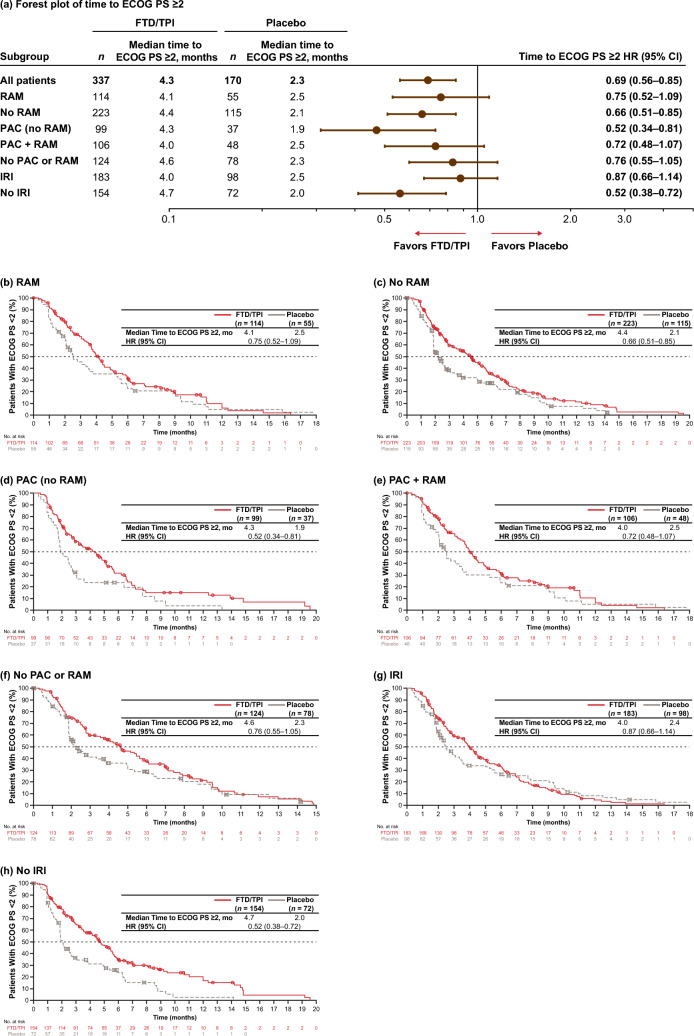
**a** Forest plot and **b**–**h** Kaplan–Meier plots of time to ECOG PS ≥ 2 across prior treatment subgroups (ITT population). *CI* confidence interval, *ECOG PS* Eastern Cooperative Oncology Group performance status scale, *FTD/TPI* trifluridine/tipiracil, *HR* hazard ratio, *IRI* irinotecan, *ITT* intent-to-treat, *PAC* paclitaxel, *RAM* ramucirumab

As previously reported, FTD/TPI significantly improved OS and PFS compared with placebo in the overall patient population (Shitara et al. 2018b). Regardless of prior therapy received, FTD/TPI treatment was consistently associated with OS, PFS, and time to ECOG ≥ 2 benefit versus placebo (Figs. 1, 2, 3). The HRs for OS for FTD/TPI versus placebo ranged from 0.47 to 0.88 (Fig. 1). Median OS with FTD/TPI was numerically longer among patients in the no RAM, no PAC or RAM, and no IRI subgroups (6.0–6.1 months) than in patients in the RAM, IRI, or RAM plus PAC subgroups (4.6–5.0 months).

Median PFS with FTD/TPI ranged from 1.9 to 2.3 months compared with 1.7–1.8 months with placebo across the prior therapy subgroups, with PFS HRs for FTD/TPI versus placebo ranging from 0.49 to 0.67 (Fig. 2). Similarly, median PFS with FTD/TPI was longer in the no RAM, no PAC or RAM, and no IRI subgroups (2.1–2.3 months) than in the prior RAM, IRI, or RAM plus PAC subgroups (1.9 months each).

Median time to ECOG ≥ 2 with FTD/TPI ranged from 4.0 to 4.7 months compared with 1.9–2.5 months with placebo across the prior therapy subgroups, with time to ECOG ≥ 2 HRs for FTD/TPI versus placebo ranging from 0.52 to 0.87 (Fig. 3). The median time to ECOG ≥ 2 with FTD/TPI was longer in the No RAM, No PAC or RAM, and No IRI subgroups (4.4–4.7 months) than in the prior RAM, PAC + RAM, and IRI subgroups (4.0–4.1 months).

Differences in PFS and OS HRs were most apparent in the IRI and no IRI subgroups, with those who did not previously receive IRI experiencing pronounced benefit with FTD/TPI (Figs. 1 and 2). OS HRs for FTD/TPI versus placebo were 0.88 (95% CI, 0.67–1.16) and 0.56 (95% CI, 0.41–0.79) in the IRI and no IRI subgroups, respectively. The corresponding PFS HRs were 0.67 (95% CI, 0.52–0.87) and 0.49 (95% CI, 0.36–0.67). To investigate whether the type of prior IRI regimen had an impact on these differences in survival, OS and PFS were assessed in patients who had received IRI as part of FOLFIRI and in those who received IRI as monotherapy or in combination with other agents (not FOLFIRI). Median OS trended shorter among those who received prior FOLFIRI (4.0 versus 3.4 months for FTD/TPI versus placebo; OS HR, 0.73; 95% CI, 0.49–1.11) than in those who received IRI as part of other regimens (6.1 versus 3.8 months; OS HR, 0.93; 95% CI, 0.63–1.36). Median PFS did not vary between these groups (Supplementary Fig. 2).

Accordingly, there was an apparent difference in time to ECOG ≥ 2 in the IRI and no IRI subgroups, with those who did not previously receive an IRI experiencing a benefit with FTD/TPI (Fig. 3). The corresponding HRs were 0.87 (95% CI, 0.66–1.14) and 0.52 (0.38–0.72). The most substantial apparent difference among the subgroups was for those with prior PAC but no RAM and no PAC or RAM; the corresponding time to ECOG ≥ 2 HRs were 0.52 (95% CI, 0.34–0.81) and 0.76 (95% CI, 0.55–1.05), respectively.

### Exposure

In general, FTD/TPI exposure was similar across all prior therapy subgroups (Supplementary Table 2). Mean dose intensity ranged from 146.4 mg/m^2^/week in the PAC without RAM subgroup to 150.0 mg/m^2^/week in the RAM plus PAC subgroup. Patients in most prior therapy subgroups received a median of 2 FTD/TPI cycles during the study; however, patients in the no PAC or RAM and no IRI subgroups received a median of 3 FTD/TPI cycles. Median duration of FTD/TPI treatment trended longer in the no RAM, no RAM or PAC, and no IRI subgroups (7.6–9.3 weeks) than in the RAM, RAM + PAC, and IRI subgroups (6.0–6.4 weeks). Mean cumulative doses were similar between subgroups with the same median FTD/TPI cycles received.

### Safety

The overall FTD/TPI safety profile was consistent across all prior therapy subgroups, with grade ≥ 3 AEs ranging from 76 to 83% among FTD/TPI-treated patients across subgroups (Table 3; Supplementary Tables 3 and 4). FTD/TPI-treated patients experienced hematologic AEs more frequently than placebo-treated patients, as expected, and marginal variations in hematologic AE incidences were noted in subgroups. Frequencies of any-grade neutropenia and anemia were generally higher in FTD/TPI-treated patients who did not previously receive IRI, PAC, or RAM (ranging from 55 to 59% and 47 to 50%, respectively) than in those who received RAM, IRI, and PAC + RAM (48% and 41–42%, respectively; Supplementary Table 3). However, frequencies of grade ≥ 3 hematologic AEs were consistent across the subgroups (Supplementary Table 4). Frequencies of gastrointestinal and other AEs were relatively well balanced between subgroups. In the PAC without RAM subgroup, overall incidences of nausea (32%) and decreased appetite (27%) were marginally lower than those observed in the other subgroups (34–44% and 32–39%, respectively). Treatment-related cardiac events occurred in 0–3% of patients in the FTD/TPI arms and in 0–3% of patients in the placebo arms of the subgroups; no differences were noted by prior therapy.

**Table 3:** Safety across prior treatment subgroups (AT population)

AE, *n* (%)	RAM		No RAM		PAC (no RAM)		RAM + PAC		No RAM or PAC		IRI		No IRI	
	FTD/	Placebo	FTD/	Placebo	FTD/	Placebo	FTD/	Placebo	FTD/	Placebo	FTD/	Placebo	FTD/	Placebo
	TPI		TPI		TPI		TPI		TPI		TPI		TPI	
	(*n* = 113)	(*n* = 55)	(*n* = 222)	(*n* = 113)	(*n* = 99)	(*n* = 36)	(*n* = 105)	(*n* = 48)	(*n* = 123)	(*n* = 77)	(*n* = 183)	(*n* = 97)	(*n* = 152)	(*n* = 71)
AE of any cause	111 (98)	47 (85)	215 (97)	110 (97)	97 (98)	36 (100)	103 (98)	41 (85)	118 (96)	74 (96)	177 (97)	90 (93)	149 (98)	67 (94)
Grade ≥ 3 AE of any cause	86 (76)	32 (58)	181 (82)	65 (58)	79 (80)	22 (61)	80 (76)	29 (60)	102 (83)	43 (56)	141 (77)	52 (54)	126 (83)	45 (63)
Any serious AE	40 (35)	27 (49)	103 (46)	43 (38)	45 (45)	12 (33)	38 (36)	24 (50)	58 (47)	31 (40)	77 (42)	43 (44)	66 (43)	27 (38)
AEs leading to treatment discontinuation	8 (7)	7 (13)	35 (16)	21 (19)	14 (14)	7 (19)	7 (7)	6 (12)	21 (17)	14 (18)	24 (13)	11 (11)	19 (12)	17 (24)
AEs leading to dose modification	68 (60)	17 (31)	127 (57)	20 (18)	63 (64)	7 (19)	63 (60)	14 (29)	64 (52)	13 (17)	98 (54)	26 (27)	97 (64)	11 (15)

*AE* adverse event, *AT* as-treated, *FTD/TPI* trifluridine/tipiracil, *IRI* irinotecan, *PAC* paclitaxel, *RAM* ramucirumab

Dosing modification rates due to AEs were consistent across subgroups (52–64% in FTD/TPI-treated patients). A higher proportion of treatment-related discontinuations due to AEs was noted among FTD/TPI-treated patients who had not previously received RAM (14–17% in the no RAM, PAC without RAM, and no PAC or RAM groups) than among those who had previously received RAM (7%).

## Discussion

These analyses of the TAGS study showed that FTD/TPI administered as third- or later-line treatment in patients with mGC/GEJC improved survival outcomes compared with placebo and was tolerable regardless of the type of previous therapy patients had received, although OS and PFS differences were most pronounced between those who had previously received IRI versus those who had not. The patterns of prior therapy received were balanced between the treatment arms. Disease and prior treatment characteristics were as anticipated across prior treatment subgroups.

Owing to variability in standard of care across regions, prior treatment patterns differed between patients in Europe and those in Japan (Di Bartolomeo et al. 2018; Longo et al. 2021; Komatsu et al. 2022). Most patients from Japan had received RAM or RAM + PAC, in line with recent large real-world data in Japan that showed nearly 70% of patients received taxanes plus RAM as second-line treatment (Komatsu et al. 2022). Although reports have indicated that taxanes plus RAM is the preferred second-line treatment in Europe (Di Bartolomeo et al. 2018; Longo et al. 2021), the percentage of patients from Europe who had received PAC and RAM was smaller (≈ 22%) in TAGS. Similarly, a greater proportion of patients from Japan had received IRI, and those in the no IRI group were predominantly from Europe and the United States. Despite these regional differences in prior treatment, no differences in PFS and OS were noted between Japanese or European patients in the TAGS trial and the variations in outcomes by prior therapy subgroups noted here were due to factors other than region (Shitara et al. 2018b).

Irrespective of prior therapy, FTD/TPI treatment was associated with OS, PFS, and daily functioning benefit versus placebo, which was consistent with results in the overall population (Shitara et al. 2018b). There were trends towards longer OS and PFS in patients who had not previously received IRI, PAC, or RAM than in those who had. This finding may likely be attributed to the extent of pretreatment: patients in the no RAM, PAC without RAM, no RAM or PAC, and no IRI subgroups had received fewer treatment lines than those in the other subgroups. Survival differences were most pronounced between the IRI (OS HR for FTD/TPI versus placebo, 0.88) and no IRI subgroups (OS HR, 0.56). Although post-progression treatment did not differ appreciably between these subgroups to explain the difference in OS, there were a few differences in baseline characteristics, prior treatment, and FTD/TPI exposure that should be noted. Compared with the IRI subgroup, a smaller proportion of patients in the no IRI subgroup had HER2-negative disease (52% versus 69% in the no IRI versus IRI group), a higher proportion had previously received taxanes (98% versus 85%), and patients were less heavily pretreated (37% versus 84% had received ≥ 3 prior regimens). In addition, patients in the no IRI subgroup had higher FTD/TPI treatment exposure than those in the IRI group (median treatment duration, 9.3 weeks versus 6.0 weeks, and mean cumulative dose of 2460 mg/m^2^ versus 1849 mg/m^2^).

With respect to daily functioning, as measured by ECOG ≥ 2 (patient no longer able to carry out any work activities; Oken et al. 1982), patients with prior exposure to PAC had an apparent greater benefit with FTD/TPI versus placebo (time to ECOG ≥ 2 HR for FTD/TPI versus placebo, 0.52 without RAM and 0.72 with RAM) compared with patients with no prior exposure to PAC or RAM (HR, 0.76; Fig. 3). These results suggest a possible greater benefit in daily functioning with prior PAC therapy, which prior RAM therapy may diminish (Fig. 3). As with OS and PFS, there was a pronounced smaller benefit with FTD/TPI versus placebo for patients with prior IRI therapy than those without.

Across subgroups, there were no appreciable differences in the FTD/TPI safety profile by prior therapy type. FTD/TPI was mainly associated with hematologic and gastrointestinal toxicities, which were demonstrated to be both predictable and manageable, as seen by the low rate of treatment-related discontinuations in the overall population and across the subgroups (Shitara et al. 2018b). Any differences noted in the FTD/TPI profile across subgroups, such as in hematologic toxicities, were marginal. Overall, cardiac AEs were infrequent in patients treated with FTD/TPI and did not vary by prior treatment received; this finding may be contrasted with fluoropyrimidine treatments, which have been associated with cardiac AE incidence rates of up to 20% (Depetris et al. 2018).

These data are consistent with related analyses of TAGS, in which median OS and PFS with FTD/TPI trended higher in patients who had received 2 prior therapies than in those who had received 3 or more lines of prior therapy (Tabernero et al. 2021), although survival benefit with FTD/TPI was observed with both groups compared with placebo. The FTD/TPI safety profiles in the third-line and fourth- or later-line patients showed no differences.

In addition, the trends of increased FTD/TPI efficacy in patients who did not previously receive IRI, PAC, or RAM are aligned with recent observations. Early data suggested feasibility of FTD/TPI plus IRI in an IRI-naive population (Mizukami et al. 2022) and FTD/TPI plus RAM in a RAM-naive and a RAM-treated population (Kawazoe et al. 2021). These data and results from ongoing trials (NCT04660760; NCT04808791) will clarify the use of FTD/TPI-containing combinations in patients with mGC/GEJC in earlier lines of treatment.

Data from these analyses add to existing evidence (Kato et al. 2019; Lorenzen et al. 2020; Kankeu Fonkoua et al. 2021; Komatsu et al. 2022) that the type of prior treatment can influence outcomes in patients with mGC/GEJC. Such data are important to help guide selection of optimally sequenced therapies so that patients with mGC/GEJC can experience the most gains in efficacy and daily functioning with little impact on tolerability. Future prospective trial data are needed to establish sequencing algorithms, particularly in the face of the changing treatment landscape, as immunotherapeutic regimens gain ground in the first-line setting.

### Limitations

These analyses are limited by their exploratory post hoc nature, which did not allow for detecting statistical significance in the subgroups. Except for 2 preplanned subgroups (RAM and no RAM), all others were analyzed post hoc. Our interpretation of the data and comparison between subgroups was limited by the substantial overlap between the subgroups. Prior treatments varied from one country to another owing to regional differences in recommended treatment schedules, drug registrations, etc. Also, because few patients in the TAGS trial had previously received PD-1/PD-L1 therapy, the impact of prior immunotherapy on FTD/TPI outcomes is unclear and will need to be examined in future studies.

## Conclusions

In conclusion, the results of this analysis indicate that FTD/TPI administered as third- or later-line treatment in patients with mGC/GEJC improved survival and daily functioning outcomes compared with placebo regardless of prior treatment. OS, PFS, and time to ECOG ≥ 2 trended longer in patients who had not previously received IRI, RAM, or PAC. FTD/TPI was tolerable regardless of the type of previous therapy patients had received.

## Supplementary Information

Below is the link to the electronic supplementary material.

Supplementary file1 (DOCX 178 KB)

## Data Availability

Data generated or analyzed during this study are on file with Taiho Oncology, Inc., and Taiho Pharmaceuticals Co., Ltd., and are not publicly available. Inquiries about data access should be sent to th-datasharing@taiho.co.jp**.**
