# Supplementary material for: Effects of prior therapies on outcomes with trifluridine/tipiracil in patients with metastatic gastric/gastroesophageal junction cancer in a randomized phase III trial (TAGS)
Source: J Cancer Res Clin Oncol. 2023 May 22;149(11):9361–74. doi: 10.1007/s00432-023-04813-z (PMC10374776; doi:10.1007/s00432-023-04813-z)
Supplement: Supplementary file 1 — Supplementary file1 (DOCX 178 KB) [file 432_2023_4813_MOESM1_ESM.docx]

**Supplementary Appendix**

Effects of prior therapies on outcomes with trifluridine/tipiracil in patients with metastatic gastric/gastroesophageal junction cancer in a randomized phase III trial (TAGS)

Kohei Shitara^1,2^, Ben George^3^, Julien Taieb^4^, Raghav Sundar^5,6^, Marwan G. Fakih^7^, Lukas Makris^8^, Karim A. Benhadji^9^, Michele Ghidini^10^

^1^National Cancer Center Hospital East, Kashiwa-Shi, Chiba 277-8577, Japan

^2^Nagoya University Graduate School of Medicine, Nagoya, Japan

^3^Medical College of Wisconsin, Milwaukee, WI, USA

^4^Hôpital Européen Georges Pompidou, Université Paris-Cité, SIRIC CARPEM, Paris, France

^5^National University Cancer Institute, Singapore

^6^Yong Loo Lin School of Medicine, National University of Singapore, Singapore, Singapore

^7^City of Hope Comprehensive Cancer Center, Duarte, CA, USA

^8^Stathmi, Inc., New Hope, PA, USA

^9^Taiho Oncology, Inc., Princeton, NJ, USA

^10^Azienda Ospedaliera di Cremona, Cremona, Italy

**Corresponding author:**

Kohei Shitara

Email: [kshitara@east.ncc.go.jp](mailto:kshitara@east.ncc.go.jp)

# Supplementary Table 1 Patient disposition by prior therapy subgroup (AT population)

|  | RAM | | | No RAM | | | PAC (no RAM) | | | RAM + PAC | | | No RAM or PAC | | | IRI | | No IRI | |
| --- | --- | --- | --- | --- | --- | --- | --- | --- | --- | --- | --- | --- | --- | --- | --- | --- | --- | --- | --- |
|  | FTD/  TPI (*n* = 113) | Placebo  (*n* = 55) | FTD/  TPI (*n* = 222) | | Placebo  (*n* = 113) | FTD/  TPI (*n* = 99) | | Placebo  (*n* = 36) | FTD/  TPI (*n* = 105) | | Placebo  (*n* = 48) | FTD/  TPI (*n* = 123) | | Placebo  (*n* = 77) | FTD/  TPI (*n* = 183) | | Placebo  (*n* = 97) | FTD/  TPI  (*n* = 152) | Placebo  (*n* = 71) |
| Ongoing study treatment, *n* (%) | 3 (3) | 0 | 16 (7) | | 3 (3) | 7 (7) | | 0 | 3 (3) | | 0 | 9 (7) | | 3 (4) | 4 (2) | | 2 (2) | 15 (10) | 1 (1) |
| Discontinued study treatment, *n* (%)  AEs  Clinical progression  Radiological progression  Patient withdrew consent  Physician’s decision  Pregnancy  Death  Other  Protocol violation | 110 (97)  3 (3)  20 (18)  82 (73)  4 (4)  1 (1)  0  0  0  0 | 55 (100)  1 (2)  11 (20)  41 (75)  0  2 (4)  0  0  0  0 | 206 (93)  30 (14)  34 (15)  110 (50)  10 (5)  10 (5)  0  11 (5)  1 (< 1)  1 (< 1) | | 110 (97)  10 (9)  24 (21)  69 (61)  4 (4)  1 (1)  0  2 (2)  0  0 | 92 (93)  12 (12)  13 (13)  58 (59)  2 (2)  4 (4)  0  2 (2)  1 (1)  1 (1) | | 36 (100)  2 (6)  12 (33)  22 (61)  0  0  0  0  0  0 | 102 (97)  2 (2)  20 (19)  76 (72)  3 (3)  1 (1)  0  0  0  0 | | 48 (100)  1 (2)  9 (19)  37 (77)  0  1 (2)  0  0  0  0 | 114 (93)  18 (15)  21 (17)  52 (42)  8 (7)  6 (5)  0  9 (7)  0  0 | | 74 (96)  8 (10)  12 (16)  47 (61)  4 (5)  1 (1)  0  2 (3)  0  0 | 179 (98)  17 (9)  31 (17)  110 (60)  9 (5)  5 (3)  0  7 (4)  0  0 | | 95 (98)  5 (5)  17 (18)  66 (68)  2 (2)  3 (3)  0  2 (2)  0  0 | 137 (90)  16 (11)  23 (15)  82 (54)  5 (3)  6 (4)  0  4 (3)  1 (1)  1 (1) | 70 (99)  6 (8)  18 (25)  44 (62)  2 (3)  0  0  0  0  0 |

*AE* adverse event, *AT* as-treated, *FTD/TPI* trifluridine/tipiracil, *IRI* irinotecan, *PAC* paclitaxel, *RAM* ramucirumab

# Supplementary Table 2 Treatment exposure across prior treatment subgroups (AT population)

|  | RAM | | | No RAM | | | PAC (no RAM) | | | RAM + PAC | | | No RAM or PAC | | | IRI | | | No IRI | |
| --- | --- | --- | --- | --- | --- | --- | --- | --- | --- | --- | --- | --- | --- | --- | --- | --- | --- | --- | --- | --- |
|  | FTD/  TPI (*n* = 113) | Placebo  (*n* = 55) | FTD/  TPI (*n* = 222) | | Placebo  (*n* = 113) | FTD/  TPI (*n* = 99) | | Placebo  (*n* = 36) | FTD/  TPI (*n* = 105) | | Placebo  (*n* = 48) | FTD/  TPI (*n* = 123) | | Placebo  (*n* = 77) | FTD/  TPI (*n* = 183) | | Placebo  (*n* = 97) | FTD/  TPI  (*n* = 152) | | Placebo  (*n* = 71) |
| Median (range) cycles initiated per patient | 2  (1–12) | 2  (1–6) | 2  (1–14) | | 2  (1–16) | 2  (1–14) | | 2  (1–5) | 2  (1–12) | | 2  (1–6) | 3  (1–12) | | 2  (1–16) | 2  (1–11) | | 2  (1–16) | 3  (1–14) | | 2  (1–10) |
| Median (range) duration of treatment, wk | 6.4  (0.7–48.4) | 5.7  (0.7–24.1) | 7.6  (0.4–62.7) | | 5.7  (0.1–63.0) | 6.7  (0.6–62.7) | | 5.7  (0.1–17.7) | 6.3  (0.7–48.4) | | 5.7  (0.7–24.1) | 8.9  (0.4–52.4) | | 5.7  (0.7–63.0) | 6.0  (0.4–47.9) | | 5.7  (0.1–63.0) | 9.3  (0.6–62.7) | | 5.7  (0.7–38.1) |
| Median (range) dose intensity, mg/m^2^/wk | 155.0  (32.4–176.1) | 163.9 (79.5–179.6) | 159.0  (26.2–177.5) | | 167.1  (17.1–191.9) | 155.5  (43.1–176.6) | | 166.5 (17.1–177.5) | 156.4 (32.4–176.1) | | 164.1  (79.5–179.1) | 159.9 (26.2–177.5) | | 167.6 (51.9–191.9) | 160.6 (26.2–177.5) | | 164.3  (17.1–181.2) | 154.6  (62.2–175.4) | | 167.7  (82.7–191.9) |
| Mean (SD) dose intensity, mg/m^2^/wk | 149.5  (23.1) | 150.2  (28.6) | 147.5  (28.5) | | 157.3  (27.4) | 146.4  (28.6) | | 154.1  (31.9) | 150.0  (23.5) | | 148.3  (29.7) | 148.5  (28.5) | | 158.8  (25.1) | 149.0  (28.8) | | 151.1  (31.1) | 147.2  (24.3) | | 160.2  (21.9) |
| Mean (SD) cumulative dose, mg/m^2^ | 1997.8 (1582.7) | 1244.3 (657.7) | 2192.7 (1685.2) | | 1642.4 (1535.6) | 2056.8 (1700.0) | | 1353.7 (734.1) | 2007.6 (1637.7) | | 1200.3 (553.1) | 2302.0 (1672.0) | | 1777.4 (1780.2) | 1849.4 (1339.5) | | 1447.1 (1423.5) | 2460.1 (1913.4) | | 1600.8 (1182.1) |

*AT* as-treated, *FTD/TPI* trifluridine/tipiracil, *IRI* irinotecan, *PAC* paclitaxel, *RAM* ramucirumab, *SD* standard deviation

# Supplementary Table 3 Most common any-grade AEs (≥ 10% of patients) in prior treatment subgroups (AT population)

| AE, *n* (%) | RAM | | No RAM | | PAC (no RAM) | | RAM + PAC | | No RAM or PAC | | IRI | | No IRI | |
| --- | --- | --- | --- | --- | --- | --- | --- | --- | --- | --- | --- | --- | --- | --- |
|  | FTD/  TPI (*n* = 113) | Placebo  (*n* = 55) | FTD/  TPI (*n* = 222) | Placebo  (*n* = 113) | FTD/  TPI (*n* = 99) | Placebo  (*n* = 36) | FTD/  TPI (*n* = 105) | Placebo  (*n* = 48) | FTD/  TPI (*n* = 123) | Placebo  (*n* = 77) | FTD/  TPI (*n* = 183) | Placebo  (*n* = 97) | FTD/  TPI  (*n* = 152) | Placebo  (*n* = 71) |
| Hematologic |  |  |  |  |  |  |  |  |  |  |  |  |  |  |
| Neutropenia^a^ | 54 (48) | 1 (2) | 122 (55) | 6 (5) | 51 (52) | 0 | 50 (48) | 1 (2) | 71 (58) | 6 (8) | 87 (48) | 5 (5) | 89 (59) | 2 (3) |
| Anemia^b^ | 46 (41) | 9 (16) | 104 (47) | 23 (20) | 42 (42) | 5 (14) | 43 (41) | 7 (15) | 62 (50) | 18 (23) | 77 (42) | 17 (18) | 73 (48) | 15 (21) |
| Leukopenia^c^ | 28 (25) | 0 | 50 (22) | 3 (3) | 16 (16) | 0 | 27 (26) | 0 | 34 (28) | 3 (4) | 33 (18) | 2 (2) | 45 (30) | 1 (1) |
| Thrombocytopenia^d^ | 17 (15) | 0 | 43 (19) | 8 (7) | 14 (14) | 2 (6) | 17 (16) | 0 | 29 (24) | 6 (8) | 28 (15) | 4 (4) | 32 (21) | 4 (6) |
| Lymphopenia | 5 (4) | 0 | 15 (7) | 8 (7) | 2 (2) | 0 | 5 (5) | 0 | 13 (11) | 8 (10) | 7 (4) | 4 (4) | 13 (9) | 4 (6) |
| Gastrointestinal |  |  |  |  |  |  |  |  |  |  |  |  |  |  |
| Nausea | 41 (36) | 13 (24) | 83 (37) | 40 (35) | 44 (44) | 17 (47) | 36 (34) | 10 (21) | 39 (32) | 23 (30) | 67 (37) | 26 (27) | 57 (38) | 27 (38) |
| Vomiting | 23 (20) | 9 (16) | 60 (27) | 25 (22) | 27 (27) | 9 (25) | 21 (20) | 9 (19) | 33 (27) | 16 (21) | 46 (25) | 20 (21) | 37 (24) | 14 (20) |
| Diarrhea | 21 (19) | 7 (13) | 55 (25) | 17 (15) | 25 (25) | 5 (14) | 21 (20) | 7 (15) | 30 (24) | 12 (16) | 42 (23) | 15 (15) | 34 (22) | 9 (13) |
| Abdominal pain | 20 (18) | 12 (22) | 35 (16) | 19 (17) | 18 (18) | 8 (22) | 18 (17) | 11 (23) | 17 (14) | 11 (14) | 33 (18) | 16 (16) | 22 (14) | 15 (21) |
| Constipation | 14 (12) | 6 (11) | 31 (14) | 19 (17) | 15 (15) | 9 (25) | 14 (13) | 5 (10) | 16 (13) | 10 (13) | 25 (14) | 15 (15) | 20 (13) | 10 (14) |
| Ascites | 5 (4) | 5 (9) | 14 (6) | 11 (10) | 5 (5) | 4 (11) | 4 (4) | 4 (8) | 9 (7) | 7 (9) | 8 (4) | 8 (8) | 11 (7) | 8 (11) |
| Dysphagia | 5 (4) | 3 (5) | 15 (7) | 5 (4) | 8 (8) | 4 (11) | 5 (5) | 3 (6) | 7 (6) | 1 (1) | 9 (5) | 6 (6) | 11 (7) | 2 (3) |
| Abdominal pain upper | 3 (3) | 3 (5) | 19 (9) | 12 (11) | 5 (5) | 4 (11) | 3 (3) | 2 (4) | 14 (11) | 8 (10) | 8 (4) | 6 (6) | 14 (9) | 9 (13) |
| Other |  |  |  |  |  |  |  |  |  |  |  |  |  |  |
| Decreased appetite | 43 (38) | 17 (31) | 72 (32) | 35 (31) | 39 (39) | 14 (39) | 41 (39) | 16 (33) | 33 (27) | 21 (27) | 63 (34) | 32 (33) | 52 (34) | 20 (28) |
| Fatigue | 28 (25) | 8 (15) | 61 (27) | 27 (24) | 27 (27) | 9 (25) | 26 (25) | 7 (15) | 34 (28) | 18 (23) | 51 (28) | 21 (22) | 38 (25) | 14 (20) |
| Pyrexia | 14 (12) | 3 (5) | 11 (5) | 5 (4) | 8 (8) | 2 (6) | 13 (12) | 2 (4) | 3 (2) | 3 (4) | 13 (7) | 6 (6) | 12 (8) | 2 (3) |
| Asthenia | 12 (11) | 7 (13) | 53 (24) | 33 (29) | 23 (23) | 18 (50) | 11 (10) | 6 (12) | 30 (24) | 15 (20) | 37 (20) | 21 (22) | 28 (18) | 19 (27) |
| Back pain | 10 (9) | 2 (4) | 15 (7) | 9 (8) | 7 (7) | 4 (11) | 9 (9) | 2 (4) | 8 (7) | 5 (6) | 14 (8) | 5 (5) | 11 (7) | 6 (8) |
| Blood alkaline  phosphatase increased | 10 (9) | 4 (7) | 20 (9) | 10 (9) | 7 (7) | 4 (11) | 9 (9) | 4 (8) | 13 (11) | 6 (8) | 17 (9) | 9 (9) | 13 (9) | 5 (7) |
| General physical health deterioration | 10 (9) | 8 (15) | 13 (6) | 9 (8) | 6 (6) | 3 (8) | 9 (9) | 8 (17) | 7 (6) | 6 (8) | 17 (9) | 8 (8) | 6 (4) | 9 (13) |
| Edema peripheral | 8 (7) | 6 (11) | 9 (4) | 6 (5) | 5 (5) | 5 (14) | 7 (7) | 6 (12) | 4 (3) | 1 (1) | 12 (7) | 6 (6) | 5 (3) | 6 (8) |
| Weight decreased | 7 (6) | 3 (5) | 13 (6) | 9 (8) | 5 (5) | 0 | 7 (7) | 3 (6) | 8 (7) | 9 (12) | 8 (4) | 4 (4) | 12 (8) | 8 (11) |
| Dyspnea | 4 (4) | 5 (9) | 20 (9) | 12 (11) | 16 (16) | 3 (8) | 4 (4) | 5 (10) | 4 (3) | 9 (12) | 12 (7) | 10 (10) | 12 (8) | 7 (10) |
| Hyponatremia | 2 (2) | 6 (11) | 3 (1) | 2 (2) | 1 (1) | 0 | 2 (2) | 5 (10) | 2 (2) | 2 (3) | 3 (2) | 7 (7) | 2 (1) | 1 (1) |

*AE* adverse event, *AT* as-treated, *FTD/TPI* trifluridine/tipiracil, *IRI* irinotecan, *PAC* paclitaxel, *RAM* ramucirumab

^a^Neutropenia and/or decreased neutrophil count

^b^Anemia and/or hemoglobin count decreased

^c^Leukopenia and/or decreased white blood cell count

^d^Thrombocytopenia and/or decreased platelet count

# Supplementary Table 4 Most common grade ≥ 3 AEs (≥ 5% of patients) in prior treatment subgroups (AT population)

| AE, *n* (%) | RAM | | No RAM | | PAC (no RAM) | | RAM + PAC | | No RAM or PAC | | IRI | | No IRI | |
| --- | --- | --- | --- | --- | --- | --- | --- | --- | --- | --- | --- | --- | --- | --- |
|  | FTD/  TPI (*n* = 113) | Placebo  (*n* = 55) | FTD/  TPI (*n* = 222) | Placebo  (*n* = 113) | FTD/  TPI (*n* = 99) | Placebo  (*n* = 36) | FTD/  TPI (*n* = 105) | Placebo  (*n* = 48) | FTD/  TPI (*n* = 123) | Placebo  (*n* = 77) | FTD/  TPI (*n* = 183) | Placebo  (*n* = 97) | FTD/  TPI  (*n* = 152) | Placebo  (*n* = 71) |
| Hematologic |  |  |  |  |  |  |  |  |  |  |  |  |  |  |
| Neutropenia^a^ | 43 (38) | 0 | 71 (32) | 0 | 32 (32) | 0 | 39 (37) | 0 | 39 (32) | 0 | 61 (33) | 0 | 53 (35) | 0 |
| Anemia^b^ | 20 (18) | 6 (11) | 44 (20) | 7 (6) | 26 (26) | 3 (8) | 18 (17) | 5 (10) | 18 (15) | 4 (5) | 37 (20) | 9 (9) | 27 (18) | 4 (6) |
| Leukopenia^c^ | 10 (9) | 0 | 21 (9) | 0 | 9 (9) | 0 | 9 (9) | 0 | 12 (10) | 0 | 13 (7) | 0 | 18 (12) | 0 |
| Gastrointestinal |  |  |  |  |  |  |  |  |  |  |  |  |  |  |
| Abdominal pain | 7 (6) | 6 (11) | 7 (3) | 9 (8) | 5 (5) | 4 (11) | 5 (5) | 6 (12) | 2 (2) | 5 (6) | 10 (5) | 7 (7) | 4 (3) | 8 (11) |
| Ascites | 3 (3) | 5 (9) | 9 (4) | 6 (5) | 3 (3) | 3 (8) | 2 (2) | 4 (8) | 6 (5) | 3 (4) | 7 (4) | 6 (6) | 5 (3) | 5 (7) |
| Nausea | 3 (3) | 3 (5) | 7 (3) | 2 (2) | 3 (3) | 1 (3) | 2 (2) | 2 (4) | 4 (3) | 1 (1) | 6 (3) | 2 (2) | 4 (3) | 3 (4) |
| Vomiting | 3 (3) | 0 | 9 (4) | 3 (3) | 5 (5) | 0 | 2 (2) | 0 | 4 (3) | 3 (4) | 6 (3) | 1 (1) | 6 (4) | 2 (3) |
| Dysphagia | 2 (2) | 1 (2) | 5 (2) | 3 (3) | 3 (3) | 2 (6) | 2 (2) | 1 (2) | 2 (2) | 1 (1) | 3 (2) | 3 (3) | 4 (3) | 1 (1) |
| Gastric hemorrhage | 0 | 3 (5) | 3 (1) | 1 (1) | 2 (2) | 1 (3) | 0 | 2 (4) | 1 (1) | 0 | 1 (1) | 2 (2) | 2 (1) | 2 (3) |
| Other |  |  |  |  |  |  |  |  |  |  |  |  |  |  |
| Decreased appetite | 13 (12) | 4 (7) | 16 (7) | 7 (6) | 12 (12) | 1 (3) | 13 (12) | 4 (8) | 4 (3) | 6 (8) | 18 (10) | 5 (5) | 11 (7) | 6 (8) |
| General physical health deterioration | 10 (9) | 8 (15) | 12 (5) | 7 (6) | 5 (5) | 3 (8) | 9 (9) | 8 (17) | 7 (6) | 4 (5) | 17 (9) | 6 (6) | 5 (3) | 9 (13) |
| Fatigue | 3 (3) | 1 (2) | 20 (9) | 9 (8) | 11 (11) | 3 (8) | 3 (3) | 1 (2) | 9 (7) | 6 (8) | 11 (6) | 4 (4) | 12 (8) | 6 (8) |
| Asthenia | 1 (1) | 2 (4) | 15 (7) | 9 (8) | 6 (6) | 6 (17) | 1 (1) | 2 (4) | 9 (7) | 3 (4) | 11 (6) | 4 (4) | 5 (3) | 7 (10) |
| Gamma-glutamyltransferase increased | 1 (1) | 4 (7) | 2 (1) | 1 (1) | 1 (1) | 0 | 1 (1) | 4 (8) | 1 (1) | 1 (1) | 3 (2) | 3 (3) | 0 | 2 (3) |
| Hyponatremia | 1 (1) | 5 (9) | 3 (1) | 2 (2) | 1 (1) | 0 | 1 (1) | 4 (8) | 2 (2) | 2 (3) | 2 (1) | 6 (6) | 2 (1) | 1 (1) |

*AE* adverse event, *AT* as-treated, *FTD/TPI* trifluridine/tipiracil, *IRI* irinotecan, *PAC* paclitaxel, *RAM* ramucirumab

^a^Neutropenia and/or decreased neutrophil count

^b^Anemia and/or hemoglobin count decreased

^c^Leukopenia and/or decreased white blood cell count

Supplementary Fig. 1 Subgroups by prior therapy (ITT population). Percentages of patients in the FTD/TPI (*n* = 337) and placebo (*n* = 170) arms.

*FTD/TPI* trifluridine/tipiracil, *IRI* irinotecan, *ITT* intent-to-treat, *PAC* paclitaxel, *RAM* ramucirumab

Supplementary Fig. 2 Kaplan–Meier plots of (A-B) OS and (C-D) PFS by prior FOLFIRI and IRI therapy (ITT population). *FOLFIRI* leucovorin, fluorouracil, and irinotecan, *FTD/TPI* trifluridine/tipiracil, *IRI* irinotecan, *ITT* intent-to-treat, *OS* overall survival, *PFS* progression-free survival

**Supplementary Fig. 1**


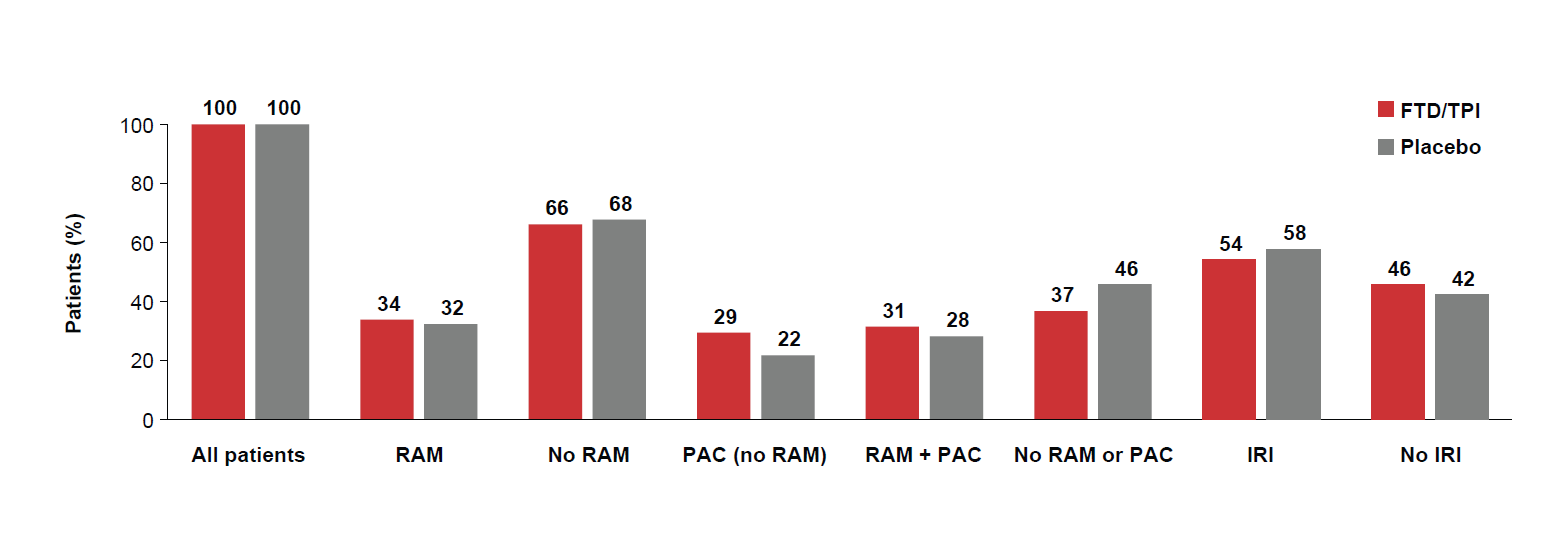


**Supplementary Fig. 2**

**
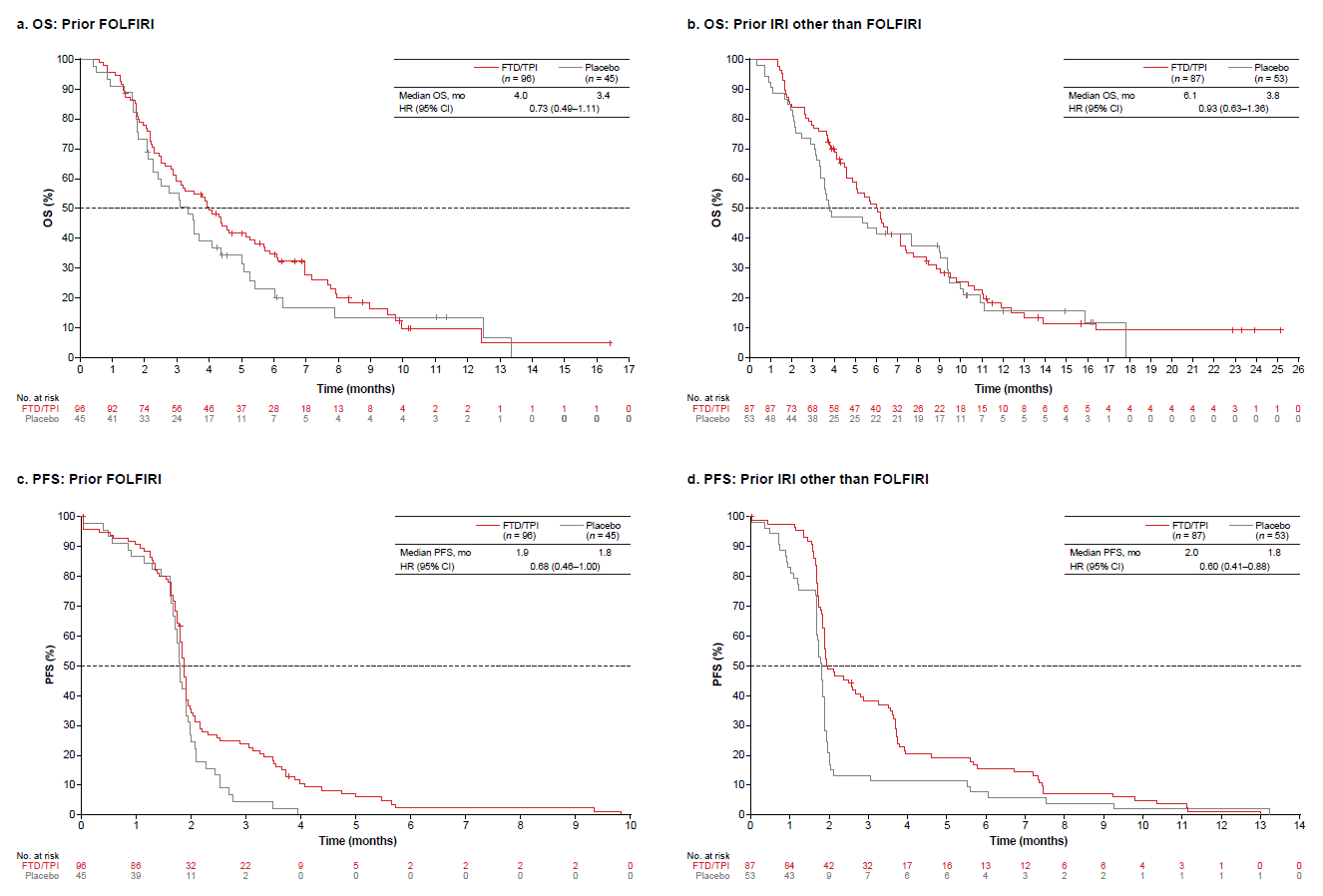
**
